# Application of preoperative CT texture analysis in papillary gastric adenocarcinoma

**DOI:** 10.1186/s12885-022-10261-8

**Published:** 2022-11-10

**Authors:** Mengying Xu, Xiangmei Qiao, Lin Li, Song Liu, Zhengyang Zhou

**Affiliations:** 1 Department of Radiology, Nanjing Drum Tower Hospital, The Affiliated Hospital of Nanjing University Medical School, No.321, Zhongshan Road, Nanjing City, 210008 Jiangsu Province China; 2 Department of Ultrasound, Nanjing Drum Tower Hospital Clinical College of Nanjing Medical University, Nanjing, 210008 China; 3 Department of Pathology, Nanjing Drum Tower Hospital, The Affiliated Hospital of Nanjing University Medical School, Nanjing, 210008 China

**Keywords:** Stomach neoplasm, Papillary adenocarcinoma, Tomography, X-ray computed, Texture analysis

## Abstract

**Background:**

This study aimed to analyze the ability of computed tomography (CT) texture analysis to discriminate papillary gastric adenocarcinoma (PGC) and to explore the diagnostic efficacy of multivariate models integrating clinical information and CT texture parameters for discriminating PGCs.

**Methods:**

This retrospective study included 20 patients with PGC and 80 patients with tubular adenocarcinoma (TAC). The clinical data and CT texture parameters based on the arterial phase (AP) and venous phase (VP) of all patients were collected and analyzed. Two CT signatures based on the AP and VP were built with the optimum features selected by the least absolute shrinkage and selection operator method. The performance of CT signatures was tested by regression analysis. Multivariate models based on regression analysis and the support vector machine (SVM) algorithm were established. The diagnostic performance of the established nomogram based on regression analysis was evaluated by receiver operating characteristic curve analysis.

**Results:**

Thirty-two and fifteen CT texture parameters extracted from AP and VP CT images, respectively, differed significantly between PGCs and TACs (all *p* < 0.05). The diagnostic performance of CT signatures based on the AP and VP achieved AUCs of 0.873 and 0.859 in distinguishing PGCs. Multivariate models that integrated two CT signatures and age based on regression analysis and the SVM algorithm showed favorable performance in preoperatively predicting PGCs (AUC = 0.922 and 0.914, respectively).

**Conclusion:**

CT texture analysis based multivariate models could preoperatively predict PGCs with satisfactory diagnostic efficacy.

**Supplementary Information:**

The online version contains supplementary material available at 10.1186/s12885-022-10261-8.

## Background

Gastric cancer (GC) is a common malignant tumor of the gastrointestinal tract and is the fifth most common tumor and the third leading cause of cancer-related deaths worldwide [1]. GC is known to have high heterogeneity and is classified into several histological subtypes according to the World Health Organization (WHO) classification system [2]. Moreover, there are differences in biological characteristics and prognosis among the different subtypes [3–6]. Papillary gastric adenocarcinoma (PGC) is one of the histological subtypes of GC and is defined as the differentiated type in the Japanese classification system [2, 7]. Previous studies suggested that the differentiated subtypes of GCs usually behaved with lower malignant potential and relatively better prognosis [8–10]. However, many studies have indicated that PGCs have malignant potential and a higher rate of lymphovascular invasion, lymph node metastasis as well as poorer prognosis [11–14]. Therefore, preoperatively discriminating PGCs is of importance for clinical treatment decision-making and prognosis estimation.

Contrast-enhanced computed tomography (CT) is the most commonly utilized imaging tool in the diagnosis, staging, and efficacy evaluation of GCs [15–18]. In clinical practice, CT morphological characteristics and conventional CT values, including the mean, maximum, and minimum, are readily accessible. However, these conventional imaging features cannot reflect the invisible characteristics of tumors. Radiomics is a noninvasive approach that allows the extraction of quantitative and high-dimensional information from medical images for data analysis. It has been widely used in numerous studies for assessing the staging, therapeutic response, and prognosis of GCs [16, 19–21]. However, radiomics has not been widely used in clinical practice, which may due to its complex and hard-to-interpret parameters.

In contrast to radiomics, CT texture analysis with relatively classic and simplified features can also quantify the mineable data of medical images [22, 23]. In addition, as an uncommon histological subtype of GC [2], the sample size of PGCs may be limited. However, radiomics analysis is commonly utilized in relatively large sample size studies [18, 21, 24]. Additionally, it may lead to the overfitting problem of model building process in the analysis of PGCs because of its limited sample size. Therefore, CT texture analysis with relatively simplified parameters may have certain advantages in the quantitative analysis of PGCs in clinical applications.

Therefore, the purpose of this study was to retrospectively analyze the ability of CT texture analysis to discriminate PGCs and to explore the diagnostic efficacy of multivariate models integrating clinical information and CT texture signatures for predicting PGCs preoperatively.

## Materials and methods

This retrospective study was approved by the Ethical Committee of Nanjing Drum Tower Hospital (Approval Documents Number: 2020–032-01). The requirement for informed consent was waived.

### Patients

Between January 2017 and August 2021, twenty patients with PGC verified by operative pathology were consecutively enrolled in the study by searching electronic radiologic image archives at our hospital. In addition, eighty consecutive patients with tubular adenocarcinoma (TAC) were consecutively enrolled in this study between April 2019 and June 2020. The following inclusion criteria were applied: (1) pathologically diagnosed as PGC and TAC postoperatively guided by the criteria of the 5th WHO Classification of Tumors of the Digestive System (2019 version) [2] and (2) abdominal contrast-enhanced CT examination performed within 2 weeks before surgery. The following exclusion criteria were applied: (1) history of preoperative treatment for GC; (2) insufficient distention of the stomach cavity; (3) poor imaging quality because of the respiratory movement or gastrointestinal peristalsis; (4) poor visibility on CT images because of the small size of GC (long diameter < 1 cm); and (5) unclear boundary of the GC lesion.

Finally, a total of one hundred patients with GC (male, 76; female, 24; median age, 66 years; age range, 31–86 years) were included. In addition, the clinical information of all patients, including residential regions of patients, body mass index (BMI), preoperative hemoglobin (Hb) concentration, history of smoking or drinking, family history, and comorbidities (diabetes, hypertension, coronary heart disease, and hepatitis), were also collected retrospectively. The details of the clinical data collection and process are listed in Additional file 1. The demographic data and clinicopathological information of all patients are shown in Table 1.

**Table 1:** Demographic data and clinicopathological information of papillary gastric adenocarcinoma and tubular adenocarcinoma

Characteristics	Papillary gastric adenocarcinoma (*n* = 20)	Tubular adenocarcinoma (*n* = 80)	*p*
Demographic data			
Gender			0.775
Male	16	60	
Female	4	20	
Age (y)			0.003*
< 60	0	24	
≥ 60	20	56	
Clinical information			
BMI (kg/m^2^)	22.71 ± 3.47	23.81 ± 3.35	0.195
Hb (g/L)	119.20 ± 18.17	128.25 ± 23.01	0.061
Residential regions			0.198
Northern China	0	9	
Southern China	20	71	
Smoking history			0.648
Absent	14	60	
Present	6	20	
Drinking history			0.287
Absent	16	71	
Present	4	9	
Family history			0.053
Absent	17	78	
Present	3	2	
Comorbidities			
Diabetes			0.147
Absent	15	71	
Present	5	9	
Hypertension			0.673
Absent	14	52	
Present	6	28	
Coronary heart disease			0.597
Absent	18	76	
Present	2	4	
Hepatitis			0.625
Absent	18	75	
Present	2	5	
Postoperative histopathological information			
T stages			0.538
1	1	8	
2	3	21	
3	15	43	
4	1	8	
N stages			0.924
N0	8	35	
N1	4	18	
N2	4	13	
N3	4	14	
Overall stages			0.812
I	3	17	
II	9	37	
III	8	25	
IV	0	1	
Differentiation degree			0.033*
Moderate & Well	16	43	
Poor	4	37	

Quantitative variables are presented as the mean ± standard deviation

*BMI* Body mass index, *Hb* Hemoglobin

*p < 0.05with chi-square test or Fisher's exact test (*n* < 5) in categorical variables and with Student’s t-test or Mann–Whitney U test in quantitative variables

#### CT acquisition parameters

Contrast-enhanced abdominal CT was performed using two 64-row scanners (uCT 780, United Imaging, Shanghai, China; Revolution Maxima, GE Healthcare, Beijing, China) and one 128-row scanner (iCT 256, Philips, Amsterdam, the Netherlands). All patients had been fasted for no less than 6 h and drank 600–1000 mL of warm water before CT examination covering the upper or the entire abdomen. Following the unenhanced scan, patients were infused with 1.5 mL/kg iodinated contrast agent (Omnipaque 350 mg I/mL, GE Healthcare) into the antecubital vein by a high-pressure syringe at a rate of 3 mL/s. Then, 30–40 s [arterial phase (AP)] and 65–70 s [venous phase (VP)] CT images were obtained. The details of the CT scan and reconstruction protocols are listed in Additional file 1.

#### CT texture analysis

**Fig. 1:**
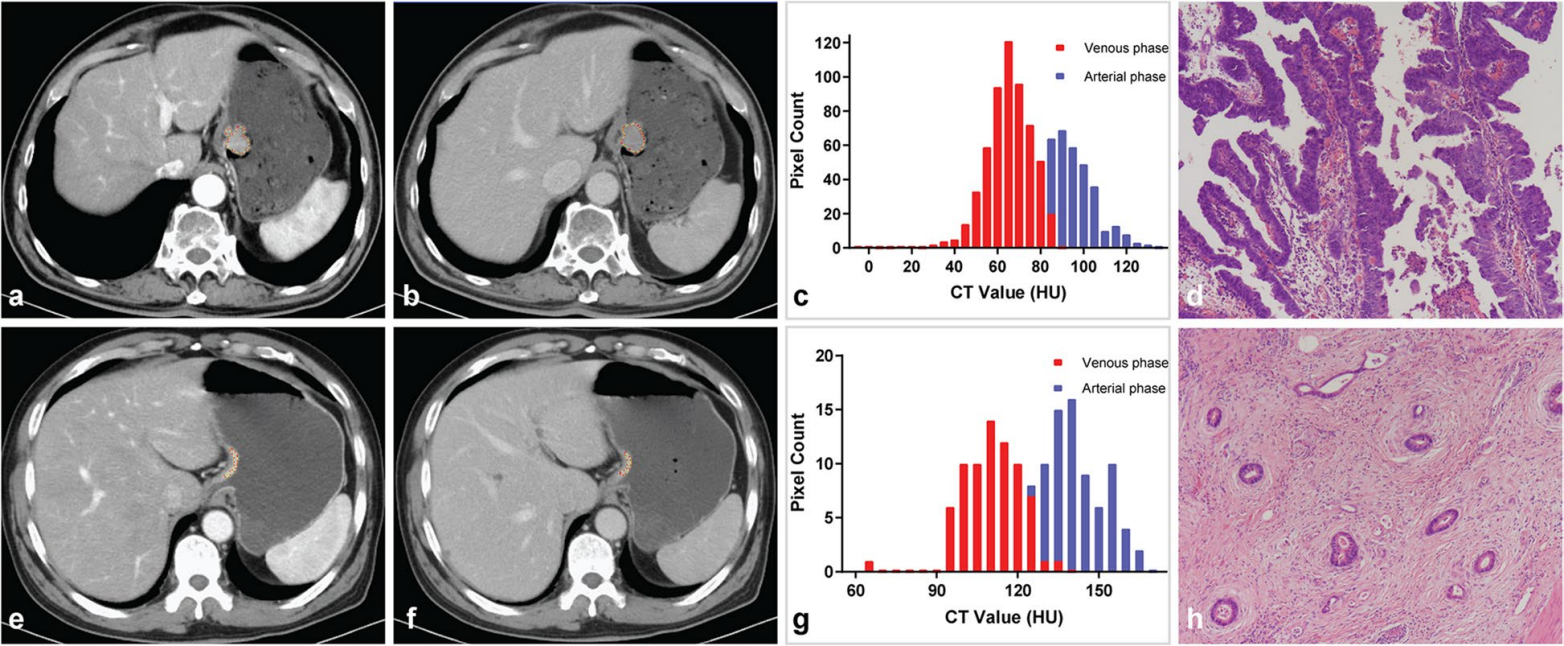
**a-d** A 67-year-old male pathologically diagnosed with PGC. **a**, **b** Polygonal regions of interest (ROIs) were manually drawn along the margin of the tumor on the largest cross-section based on the CT images of arterial and venous phases. **c** Texture parameters were extracted from the polygonal ROIs. **d** Haematoxylin and eosin (H&E) staining of the postoperative specimen (original magnification, × 100). The tumor was characterized by finger-like projections of tissues supported by central fibrovascular cores and was confirmed as PGC. **e–h** A 64-year-old male pathologically diagnosed with TAC. **e**, **f** Polygonal regions of interest (ROIs) were manually drawn along the margin of the tumor on the largest cross-section based on the CT images of arterial and venous phases. **g** Texture parameters were extracted from the polygonal ROIs. **h** H&E staining of the postoperative specimen (original magnification, × 100). The tumor showed dilated or slit-like branching tubules and was confirmed as TAC. CT, computed tomography; PGC, papillary gastric adenocarcinoma; TAC, tubular adenocarcinoma

AP and VP CT images were uploaded into in-house software (Image Analyzer 2.0). All the images were reviewed by Radiologist 1. Polygonal ROIs on AP (mean size, 347.03 mm^2^; range, 24.36–2179.76 mm^2^) and VP (mean size, 355.85 mm^2^; range, 41.12–2129.96 mm^2^) CT images were manually segmented along the margin of the tumor on the largest cross-section (Fig. 1). The normal gastric wall tissue and gastric cavity contents were avoided during the drawing of ROIs. The details of the extracted CT texture parameters are listed in Additional file 1. Texture parameters derived from ROIs delineated by Radiologist 1 were used to discriminate PGCs from TACs. Radiologist 2 repeated the above procedure to determine the interobserver reproducibility.

### Development of the multivariate model

Starting with the statistically significant (*p* < 0.05) variables of the two-phase CT texture parameters in univariate analysis, the least absolute shrinkage and selection operator (LASSO) was utilized for dimension reduction. Then, two CT signatures based on the AP and VP were established using the linear combination of the selected features weighted by their respective coefficients [25]. The multivariate model for discriminating PGCs from TACs was established by using multivariate binomial logistic regression analysis. The Hosmer–Lemeshow test was used to measure the goodness of fit. Then, a nomogram was built with both clinical features and two CT signatures using the R software package (version 3.5.2: http://www.Rproject.org). The diagnostic performance of the established model was evaluated by receiver operating characteristic (ROC) curve analysis.

In addition, another multivariate model integrating both the clinical data and two CT signatures was also established by applying the support vector machine (SVM) classifier with fivefold cross-validation.

### Pathological assessment

**Fig. 2:**
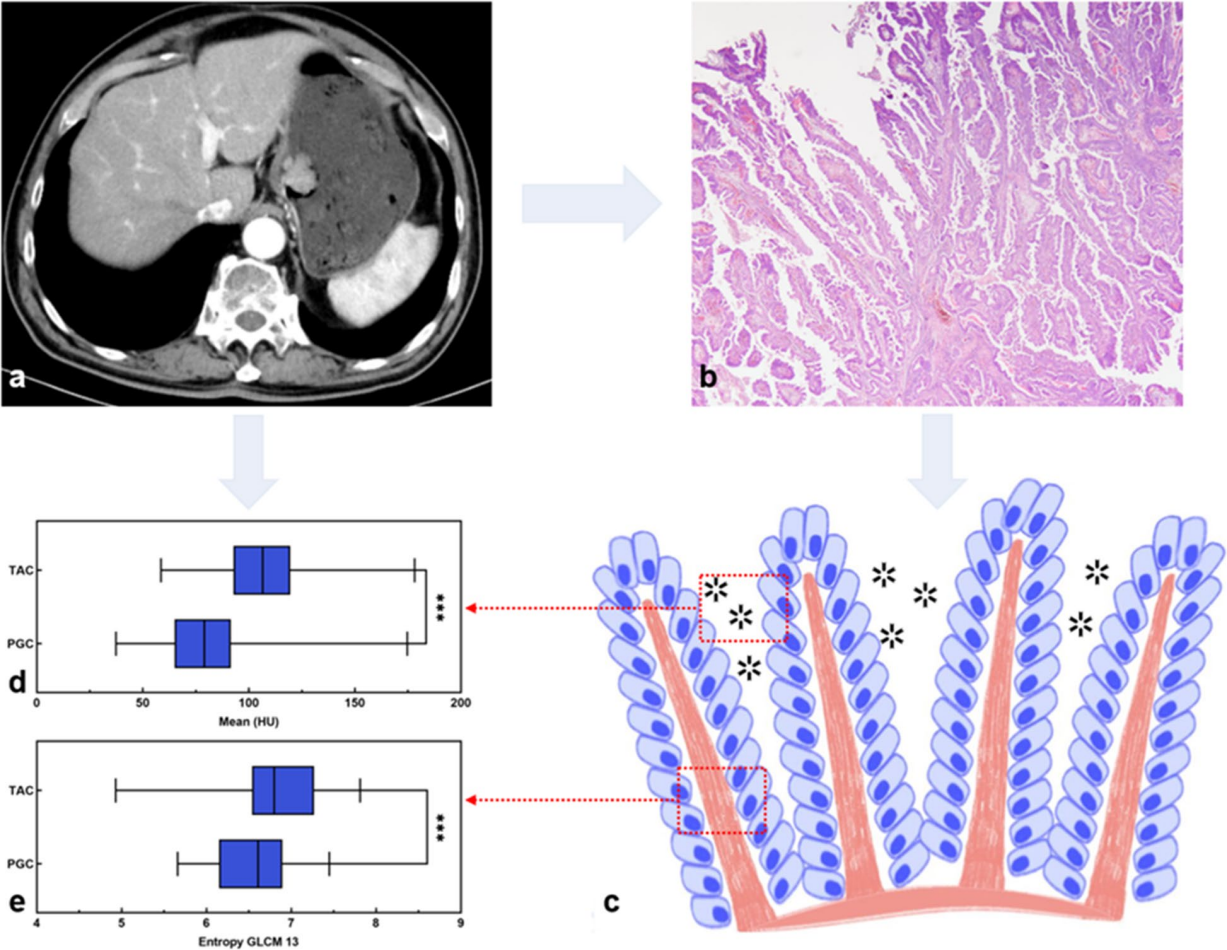
Illustration of the CT texture parameters and histopathologic features of PGCs. **a** CT image based on the arterial phase shows a mass lesion in the cardia. **b** Haematoxylin and eosin (H&E) staining of the postoperative specimen, which was pathologically diagnosed with PGC (original magnification, × 20). **c** A pathological illustration of PGC. The illustration shows that PGC is characterized by gastric adenocarcinoma with papillary epithelium featuring the central fibrovascular core with relatively larger gap within its tissue structures (asterisk). **d**, **e** Boxplot graphics show the concentration and dispersion distributions of mean and Entropy GLCM 13 based on the arterial phase CT images of the PGCs and TACs. The mean values in PGCs were significantly lower than those in TACs. This may be because PGCs composed of elongated finger-like processes have relatively larger gaps within their tissue structures and thus exhibit lower enhancement than TACs. The values of Entropy GLCM 13 in PGCs were significantly lower than those in TACs. This may be because PGCs are composed of finger-like protuberant structures with central fiber vascular bundles as the core, and tumors may be less prone to ischemic necrosis with a relatively adequate distal blood supply

After gastrectomy, all gastric specimens were processed according to standard pathological procedures. Then, the histopathological subtypes of GC were examined retrospectively by a pathologist (with eight years of experience in the diagnosis of the digestive system) guided by the 5th WHO classification criteria [2]. PGC is characterized by gastric adenocarcinoma with papillary epithelium around a central fibrovascular core (Fig. 2) and is defined as papillary structures accounting for more than 50% of the tumor area [2, 26]. TAC is characterized by dilated or slit-like branching tubules [2].

Moreover, the pathological information of each lesion, including T stage, N stage, overall stage, and differentiation degree, was also assessed and recorded.

### Statistical analysis

The chi-square or Fisher's exact test (*n* < 5) was utilized to assess the differences between PGCs and TACs in categorical variables of clinical and histopathological information. After performing the Shapiro–Wilk test for normality analysis, the differences in quantitative variables were assessed by the Mann–Whitney U test or Student’s t-test. ROC curve analysis was performed, and the area under the ROC curve (AUC), diagnostic sensitivity, specificity, and accuracy were calculated. The interobserver agreement of the CT parameters extracted by two radiologists was determined via intraclass correlation coefficient (ICC), which was classified as poor agreement (0.000–0.200), fair agreement (0.201–0.400), moderate agreement (0.401–0.600), good agreement (0.601–0.800), or excellent agreement (0.801–1.000). Statistical analyses were performed using SPSS (version 22.0 for Microsoft Windows × 64, SPSS). A two-sided *p* value less than 0.05 was considered statistically significant.

## Results

### Demographic data

The demographic data of the PGCs and TACs are summarized in Table 1. The distribution of age differed significantly between the two groups (*p* = 0.003). However, no significant difference was found in the distribution of gender between the two groups (*p* = 0.775).

### Clinical data

The clinical information of the PGCs and TACs are listed in Table 1. There were no significant differences in residential regions, BMI, Hb, history of smoking or drinking, family history, or comorbidities (all *p* > 0.05, Table 1).

### CT texture parameters

Table 2 summarizes the results of the univariate analysis for the texture parameters based on the AP CT images of the PGCs and TACs. There were significant differences in multiple parameters between the two groups (all *p* < 0.05), including the mean, standard deviation, max frequency, mode, minimum, maximum, 5^th^-90^th^ percentiles, kurtosis, area, max diameter, SsD low, histogram width, Entropy GLCM 10–11, Entropy GLCM 13, Energy GLCM 10–13, Inertia GLCM 10–13, and Variance GLCM 10–13. The diagnostic performance achieved AUCs ranging from 0.649 to 0.858 (Table A 1).

**Table 2:** Statistical description and univariate analysis of texture parameters based on the arterial phase

Parameters	Papillary gastric adenocarcinoma (*n* = 20)	Tubular adenocarcinoma (*n* = 80)	*p*
Mean (HU)	78.98 (64.93, 91.47)	106.47 (92.76, 119.55)	< 0.001*
Standard deviation	14.05 (12.33, 16.26)	17.26 (13.68, 20.52)	0.003*
Max frequency	30.50 (16.25, 44.50)	15.00 (9.00, 25.00)	0.001*
Mode (HU)	79.50 (65.00, 93.75)	102.00 (87.50, 120.00)	< 0.001*
Minimum (HU)	39.50 (17.75, 47.75)	60.00 (45.25, 74.25)	< 0.001*
Maximum (HU)	120.50 (104.75, 131.75)	151.00 (136.00, 173.50)	< 0.001*
5^th^ percentile (HU)	55.50 (40.00, 66.50)	76.00 (66.00, 90.00)	< 0.001*
10^th^ percentile (HU)	62.50 (48.25, 71.75)	80.50 (73.00, 97.75)	< 0.001*
25^th^ percentile (HU)	71.00 (57.50, 80.75)	93.00 (82.00, 109.25)	< 0.001*
50^th^ percentile (HU)	78.50 (65.25, 92.50)	106.00 (91.50, 120.00)	< 0.001*
75^th^ percentile (HU)	87.00 (74.25, 102.25)	119.00 (101.50, 131.50)	< 0.001*
90^th^ percentile (HU)	94.50 (82.00, 111.00)	128.00 (113.00, 146.50)	< 0.001*
Kurtosis	3.18 (2.91, 3.67)	2.84 (2.57, 3.22)	0.001*
Area (cm^2^)	415.27 (229.82, 689.94)	203.21 (123.81, 410.80)	0.013*
Max diameter (cm)	40.00 (28.96, 55.03)	29.48 (22.08, 37.91)	0.006*
SsD low	53.50 (30.75, 65.50)	75.50 (63.25, 96.50)	< 0.001*
Histogram width (HU)	35.00 (31.75, 39.75)	44.50 (36.00, 53.00)	0.001*
Entropy GLCM 10	6.77 (6.32, 7.14)	7.05 (6.63, 7.51)	0.038*
Entropy GLCM 11	6.61 (6.28, 6.87)	6.87 (6.54, 7.31)	0.034*
Entropy GLCM 13	6.61 (6.15, 6.89)	6.80 (6.54, 7.27)	0.040*
Energy GLCM 10 ^a^	12.45 (9.12, 16.97)	9.70 (7.11, 12.85)	0.010*
Energy GLCM 11 ^a^	13.96 (11.27, 17.56)	11.18 (8.30, 13.67)	0.005*
Energy GLCM 12 ^a^	11.31 (9.20, 15.99)	9.87 (7.13, 12.40)	0.029*
Energy GLCM 13 ^a^	14.04 (11.22, 19.31)	11.77 (8.73, 14.80)	0.009*
Inertia GLCM 10	6.29 (5.11, 9.09)	9.84 (7.88, 13.30)	< 0.001*
Inertia GLCM 11	4.95 (3.98, 6.43)	6.41 (5.29, 8.20)	0.005*
Inertia GLCM 12	8.17 (6.44, 10.81)	10.01 (8.26, 13.37)	0.031*
Inertia GLCM 13	4.47 (3.26, 6.10)	5.81 (4.70, 7.67)	0.005*
Variance GLCM 10	11.73 (8.85, 16.27)	17.19 (11.48, 25.16)	0.002*
Variance GLCM 11	11.82 (9.45, 16.65)	17.52 (11.48, 25.33)	0.003*
Variance GLCM 12	11.88 (9.08, 15.95)	17.88 (11.27, 25.05)	0.002*
Variance GLCM 13	11.88 (9.04, 16.00)	17.94 (11.43, 25.48)	0.002*

The data are presented as the median (1st quartile, 3rd quartile)

*GLCM* Gray-level cooccurrence matrix

*p < 0.05 with Mann–Whitney U test

^a^, × 10^–3^

Moreover, the mean, max frequency, mode, minimum, 25^th^-90^th^ percentiles, kurtosis, area, max diameter, SsD low, Inertia GLCM 10, and Inertia GLCM 12–13 derived from the VP CT images differed significantly between PGCs and TACs (Table 3). The corresponding AUCs ranged from 0.647 to 0.758 (Table A 2).

**Table 3:** Statistical description and univariate analysis of texture parameters based on the venous phase

Parameters	Papillary gastric adenocarcinoma (*n* = 20)	Tubular adenocarcinoma (*n* = 80)	*p*
Mean (HU)	77.29 (70.28, 82.51)	84.89 (76.69, 98.18)	0.012*
Max frequency	36.00 (20.75, 52.50)	17.00 (11.00, 29.00)	< 0.001*
Mode (HU)	76.00 (68.25, 82.00)	84.00 (71.25, 98.75)	0.029*
Minimum (HU)	42.00 (24.00, 47.75)	45.50 (37.00, 59.75)	0.042*
25^th^ percentile (HU)	70.00 (63.50, 75.00)	76.00 (66.00, 90.50)	0.041*
50^th^ percentile (HU)	77.00 (70.50, 82.00)	85.00 (76.00, 97.75)	0.019*
75^th^ percentile (HU)	84.00 (81.00, 90.00)	94.50 (86.00, 107.50)	0.007*
90^th^ percentile (HU)	92.00 (89.25, 100.00)	103.00 (93.25, 114.25)	0.006*
Kurtosis	3.20 (2.76, 3.61)	2.91 (2.65, 3.12)	0.031*
Area (cm^2^)	498.14 (235.19, 687.49)	218.81 (121.31, 367.32)	0.001*
Max diameter (cm)	44.72 (25.79, 63.60)	29.88 (22.96, 37.71)	0.002*
SsD low	52.00 (39.75, 60.00)	61.00 (51.25, 77.50)	0.005*
Inertia GLCM 10	6.68 (4.74, 8.00)	8.20 (6.61, 9.78)	0.011*
Inertia GLCM 12	6.65 (5.02, 8.03)	7.66 (6.44, 10.55)	0.035*
Inertia GLCM 13	3.64 (2.90, 4.61)	4.63 (3.55, 5.92)	0.011*

The data are presented as the median (1st quartile, 3rd quartile)

*GLCM* Gray-level cooccurrence matrix

*p < 0.05 with Mann–Whitney U test

^a^, × 10^–3^

### Development of multivariate models

#### Feature selection and construction of CT signatures

**Fig. 3:**
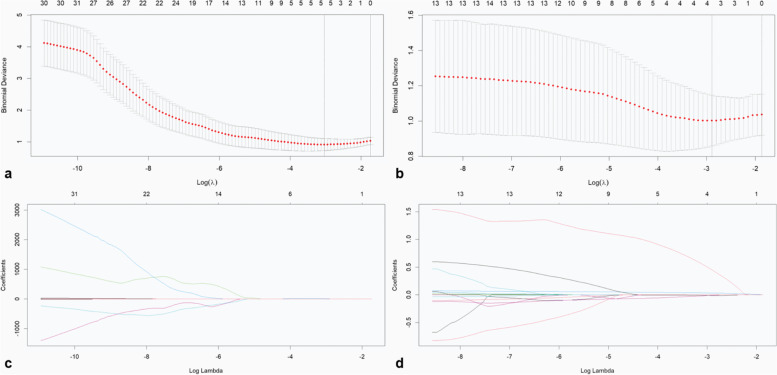
Feature selection was performed using the least absolute shrinkage and selection operator (LASSO) regression model. **a**, **b** Tuning parameter (λ) selection in the LASSO model used fivefold cross-validation via minimum criteria. Vertical lines were drawn at the optimal values using the minimum criteria and 1 standard error of the minimum criteria. For the selection of texture parameters based on the arterial phase CT images, the optimal λ value of 0.0363 with log (λ) =  − 3.3159 was chosen. For the selection of texture parameters based on the venous phase CT images, the optimal λ value of 0.0555 with log (λ) =  − 2.8914 was chosen. **c**, **d** For the selection of texture parameters based on the arterial and venous phase CT images, LASSO coefficient profiles of the 32 and 15 selected features, respectively. Two coefficient profile plots were generated versus the selected log (λ) value using fivefold cross-validation; five and four selected features with nonzero coefficients were retained

Thirty-two and fifteen CT texture parameters extracted from the AP and VP CT images, respectively, differed significantly between PGCs and TACs, and they were entered into the LASSO analysis for dimension reduction (Fig. 3). Then, two CT signatures were built using the selected optimum five and four features weighted by their respective coefficients. The diagnostic performance of the CT signatures based on the AP and VP achieved AUCs of 0.873 and 0.859, respectively, in distinguishing PGCs from TACs (Table 4).

**Table 4:** Diagnostic performance of the CT signatures and the multivariate model based on regression analysis

	Cutoff	Sensitivity	Specificity	AUC	Accuracy	*p* value
CT signature (AP)	-0.82	0.750	0.937	0.873	0.900	< 0.001*
CT signature (VP)	-1.27	0.850	0.825	0.859	0.830	< 0.001*
Multivariate model	0.27	0.900	0.900	0.922	0.900	< 0.001*

*AUC* Area under the receiver operating characteristic (ROC) curve

*AP *Arterial phase*, VP *Venous phase

*p < 0.05 with ROC curve analysis

#### Construction of multivariate models

**Fig. 4:**
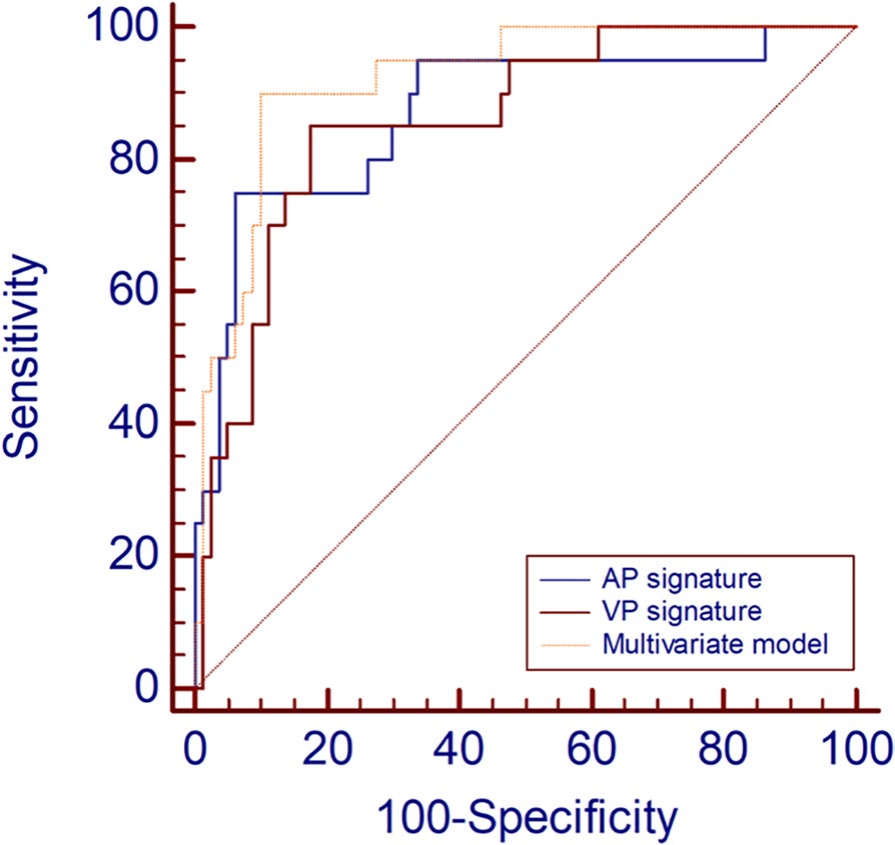
Receiver operating characteristic curve analysis based on binomial logistic regression analysis for preoperatively discriminating PGCs. The areas under the curves of the multivariate model, AP signature, and VP signature were 0.922, 0.873, and 0.859, respectively. PGC, papillary gastric adenocarcinoma; AP, arterial phase; VP, venous phase

**Fig. 5:**
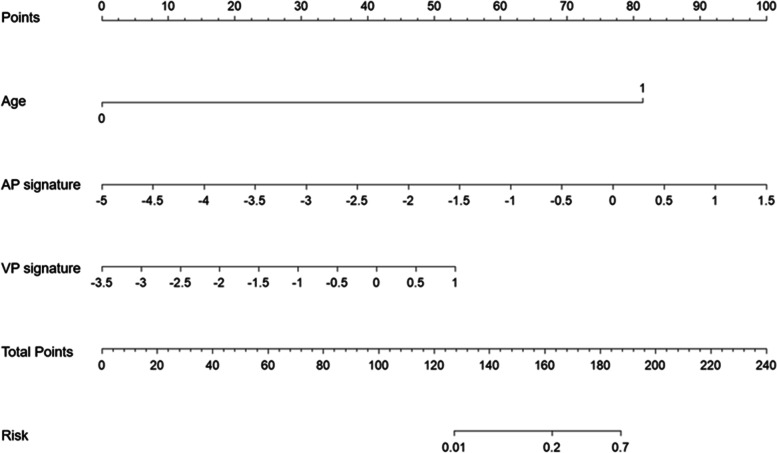
A nomogram based on the multivariate logistic regression model for discriminating PGCs from TACs integrating the features of age, AP signature, and VP signature. PGC, papillary gastric adenocarcinoma; TAC, tubular adenocarcinoma; AP, arterial phase; VP, venous phase

The multivariate model based on regression analysis that integrated age and two CT signatures further improved the diagnostic efficacy (AUC = 0.922, Table 4). The ROC curve of the multivariate model is shown in Fig. 4. The cutoff value of the multivariate model was 0.27, which yield a sensitivity, specificity, and accuracy of 90.0%, 90.0%, and 90.0%, respectively. The nomogram constructed based on the multivariate model for discriminating PGCs from TACs is displayed in Fig. 5. In addition, another multivariate model was built using the SVM algorithm, and it achieved an AUC of 0.914.

### Interobserver agreement

For the texture parameters based on the AP and VP, 34/35 and 25/35 parameters, respectively, showed good to excellent interobserver agreement (Tables A 3 and A 4).

## Discussion

PGC is an uncommon histological subtype of GC and is defined as the differentiated type [2]. However, previous studies reported that the prognosis of PGCs was poor [13, 27]. In the current study, clinicopathological information and CT texture parameters based on the AP and VP of PGCs were retrospectively analyzed and compared with those of TACs. Our study found that multiple CT texture parameters differed significantly between PGCs and TACs. Moreover, the nomogram based on the multivariate regression model also demonstrated satisfactory performance for predicting PGCs preoperatively, which helps with clinical decision-making.

CT texture analysis plays an important role in the noninvasive and quantitative evaluation of tumor heterogeneity by analyzing the intensity and spatial distribution characteristics of image pixels [22, 23, 28]. In this study, there were significant differences in 32/35 parameters based on the AP and 15/35 parameters based on the VP between the two groups. The CT scans of AP in this study were performed with a 30–40 s delay after the injection of the contrast agent, and this was performed with a 40 s delay for most of the patients (97/100). The relatively late scanning time of the AP allows more contrast agent to enter the tumor parenchyma from the large vessels [29], and, there is no contrast agent flowing into the venous system. In addition, a recent study also demonstrated that CT findings based on the 40 s AP could discriminate gastric poorly cohesive carcinoma from TAC [30]. Therefore, the CT texture parameters extracted from the AP might be more advantageous in distinguishing PGCs.

The values of texture parameters reflecting the degree of lesion enhancement in PGCs, including the mean, minimum, maximum, and 5^th^-90^th^ percentiles, were significantly lower than those in TACs. This may be because PGCs characterized by elongated finger-like structures have relatively larger gaps within their tissue structures [26]. Thus, the tumor tends to exhibit lower enhancement than TACs due to the relatively less densely arranged tumor cells. In addition, the values of standard deviation, Entropy GLCM 10–11, Entropy GLCM 13, and Variance GLCM 10–13 in PGCs were also significantly lower than those in TACs. Standard deviation and Variance GLCM reflect the dispersion of pixel gray level distributions, and Entropy GLCM reflects the complexity of pixel distributions [22, 31, 32]. Our results suggested that the enhancement in PGCs was relatively homogeneous. This may be because PGCs are composed of finger-like protuberant structures with central fiber vascular bundles as the core, and tumors with a relatively adequate distal blood supply may be less prone to ischemic necrosis. Moreover, the values of Energy GLCM indicating the uniformity of pixel distributions were higher in PGCs than in TACs [31]. The distribution of significant texture parameters derived from the VP between PGCs and TACs was similar to parameters derived from the AP.

In this study, the diagnostic efficacies of significant texture parameters extracted from the AP and VP CT images achieved AUCs varying from 0.647 to 0.858 in predicting PGCs. To explore the optimal parameters for distinguishing PGCs, LASSO analysis was utilized for dimensional reduction. Then, two CT signatures based on the AP and VP were established and analyzed by using regression analysis. The AUCs were 0.873 and 0.859 for the CT signatures based on AP and VP, respectively. In this study, the distribution of age differed significantly between the two groups. PGCs were more likely to occur in elderly patients, which was consistent with previous studies on PGCs [13, 27]. Therefore, our study further explored the diagnostic performance of multivariate models integrating age and CT signatures based on the AP and VP for predicting PGCs. The multivariate model based on the regression analysis further improved the diagnostic efficacy (AUC = 0.922). The nomogram based on the regression analysis visualized the model, which could benefit clinical applications. In addition, the SVM algorithm was also applied to establish another multivariate model and achieved favorable diagnostic performance (AUC = 0.914). PGC is an uncommon subtype of GC [2], and the sample size of PGCs enrolled in this study was relatively small. Texture analysis is more classic with fewer parameters than radiomics. However, there might be an overfitting problem in the model building process, resulting in relatively higher AUCs. Therefore, the results of this study also need to be further validated by large-scale studies. In this study, most CT texture parameters achieved good to excellent interobserver agreement.

Certain limitations in this study are worthy of consideration. First, PGC is an uncommon histological subtype of GC. The sample size of PGCs in the current study was limited. Second, it was a retrospective study from a single center, which might have resulted in sample selection bias. Therefore, the results of this study need to be further validated by multicenter large-scale studies and refined with patients from different food habits as well as different age groups. Third, CT texture parameters were derived from two-dimensional ROIs instead of three-dimensional volumes of interest, which might lead to the loss of longitudinal information. However, two-dimensional ROIs are convenient to apply in clinical practice.

In conclusion, multiple texture parameters based on the AP and VP CT images differed significantly between PGCs and TACs. The combination of age and two CT signatures could predict PGCs preoperatively with satisfactory diagnostic efficacy.

## Supplementary Information

**Additional file 1.**

**Additional file 2.**

## Data Availability

The datasets used and analyzed during the current study are available from the corresponding author on reasonable request.

## Abbreviations

AP: Arterial phase
AUC: Area under the receiver operating characteristic curve
BMI: Body mass index
CT: Computed tomography
GC: Gastric cancer
GLCM: Gray-level cooccurrence matrix
Hb: Hemoglobin
ICC: Intraclass correlation coefficient
LASSO: Least absolute shrinkage and selection operator
PGC: Papillary gastric adenocarcinoma
ROC: Receiver operating characteristic
ROI: Region of interest
SVM: Support vector machine
TAC: Tubular adenocarcinoma
VP: Venous phase
WHO: World Health Organization
