# Supplementary material for: Application of preoperative CT texture analysis in papillary gastric adenocarcinoma
Source: BMC Cancer. 2022 Nov 10;22:1161. doi: 10.1186/s12885-022-10261-8 (PMC9650902; doi:10.1186/s12885-022-10261-8)
Supplement: Supplementary file 1 — Additional file 1. [file 12885_2022_10261_MOESM1_ESM.pdf]

S1. The details of clinical data collection and process

Clinical information of all patients, including residential regions of patients, body mass index (BMI), preoperative hemoglobin (Hb) concentration, history of smoking or drinking, family history, and comorbidities (diabetes, hypertension, coronary heart disease, and hepatitis), were also collected retrospectively. In this study, all enrolled patients were from China. As an important geographical demarcation line in China, the Qinling Mountains-Huaihe River line separates the northern and southern regions of China that correspond with different food habits. Therefore, we used the distribution of patients' regions to represent the differences in patients' food habits. Moreover, the BMI data of 4 patients were missing in this retrospective study. The values of BMI fitted the normal distribution after using the Shapiro-Wilk test for normality analysis, therefore, the missing data were replaced by the mean value of BMI of the remaining patients.

S2. The details of the CT scan and reconstruction protocols

CT examination was performed using two 64-row scanners (uCT 780, United Imaging, Shanghai, China; Revolution Maxima, GE Healthcare, Beijing, China) and one 128-row scanner (iCT 256, Philips, Amsterdam, the Netherlands). In details, in this retrospective study, the number of patients performed with CT examinations on the above CT scanners were 97, 2, and 1, respectively. The proportion of patients performed with CT examinations on the scanner ((uCT 780, United Imaging, Shanghai, China) was the largest, the CT scan parameters of this scanner were as follows: tube voltage 120 kV, tube current 150-250 mA, field of view 35-50 cm, matrix 512 × 512, rotation time 0.7 s, and pitch 1.0875. CT images were reconstructed with a 1-mm section thickness for multiplanar reconstruction and with a 5-mm section thickness for the measurement of CT values due to the signal-to-noise ratio.

S3. The details of the CT texture parameters

Texture parameters were as follows: (1) the first-order features included the mean, standard deviation, max frequency, mode, minimum, maximum, cumulative percentiles (the 5th, 10th, 25th, 50th, 75th, and 90th percentiles), skewness, kurtosis, entropy, and histogram width; (2) the second-order features were from the gray-level cooccurrence matrix (GLCM) and included Entropy GLCM, Energy GLCM, Inertia GLCM, and Variance GLCM.
