# Supplementary material for: Application of preoperative CT texture analysis in papillary gastric adenocarcinoma
Source: BMC Cancer. 2022 Nov 10;22:1161. doi: 10.1186/s12885-022-10261-8 (PMC9650902; doi:10.1186/s12885-022-10261-8)
Supplement: Supplementary file 2 — Additional file 2. [file 12885_2022_10261_MOESM2_ESM.pdf]

Table A1 Diagnostic performance of the CT texture parameters based on the arterial phase

Parameters	Cutoff	Sensitivity	Specificity	AUC	Accuracy	*p*
Mean (HU)	92.41	0.800	0.750	0.832	0.760	<0.001*
Standard deviation	17.93	0.950	0.450	0.719	0.550	<0.001*
Max frequency	21.00	0.700	0.725	0.747	0.720	<0.001*
Mode (HU)	94.00	0.900	0.650	0.791	0.700	<0.001*
Minimum (HU)	49.00	0.850	0.700	0.781	0.730	<0.001*
Maximum (HU)	132.00	0.800	0.787	0.821	0.790	<0.001*
5th percentile (HU)	58.00	0.650	0.900	0.799	0.850	<0.001*
10th percentile (HU)	68.00	0.750	0.800	0.799	0.790	<0.001*
25th percentile (HU)	77.00	0.750	0.800	0.810	0.790	<0.001*
50th percentile (HU)	93.00	0.800	0.737	0.823	0.750	<0.001*
75th percentile (HU)	94.00	0.700	0.862	0.846	0.830	<0.001*
90th percentile (HU)	102.00	0.700	0.912	0.858	0.870	<0.001*
Kurtosis	2.77	0.950	0.475	0.732	0.570	<0.001*
Area (cm^2)	225.21	0.800	0.562	0.681	0.610	0.011*
Max diameter (cm)	36.97	0.650	0.750	0.698	0.730	0.008*
SsD low	56.00	0.650	0.862	0.785	0.820	<0.001*
Histogram width (HU)	45.00	0.950	0.462	0.750	0.560	<0.001*
Entropy GLCM 10	6.96	0.700	0.575	0.651	0.600	0.025*
Entropy GLCM 11	6.91	0.850	0.475	0.654	0.550	0.027*
Entropy GLCM 13	6.91	0.850	0.412	0.649	0.500	0.028*
Energy GLCM 10	11.10 [a]	0.650	0.675	0.687	0.670	0.005*
Energy GLCM 11	11.00 [a]	0.850	0.500	0.703	0.570	0.003*
Energy GLCM 12	9.00 [a]	0.850	0.437	0.658	0.520	0.021*
Energy GLCM 13	12.00 [a]	0.750	0.562	0.689	0.600	0.004*
Inertia GLCM 10	6.29	0.550	0.937	0.757	0.860	<0.001*
Inertia GLCM 11	5.20	0.600	0.775	0.706	0.740	0.006*
Inertia GLCM 12	8.21	0.550	0.762	0.657	0.720	0.036*
Inertia GLCM 13	5.15	0.750	0.700	0.704	0.710	0.007*
Variance GLCM 10	19.71	0.950	0.450	0.725	0.550	<0.001*
Variance GLCM 11	20.01	0.950	0.450	0.716	0.550	<0.001*
Variance GLCM 12	16.79	0.850	0.550	0.726	0.610	<0.001*
Variance GLCM 13	17.91	0.900	0.500	0.729	0.580	<0.001*

AUC, area under the receiver operating characteristic (ROC) curve; GLCM, gray-level cooccurrence matrix; [a], $\times 10^{-3}$; *$p<0.05$ with ROC curve analysis.

Table A2 Diagnostic performance of the CT texture parameters based on the venous phase

Parameters	Cutoff	Sensitivity	Specificity	AUC	Accuracy	*p*
Mean (HU)	82.75	0.800	0.587	0.681	0.630	0.004*
Max frequency	31.00	0.700	0.787	0.758	0.770	<0.001*
Mode (HU)	83.00	0.850	0.525	0.659	0.590	0.007*
Minimum (HU)	48.00	0.850	0.462	0.647	0.540	0.024*
25th percentile (HU)	75.00	0.800	0.537	0.648	0.590	0.023*
50th percentile (HU)	82.00	0.800	0.575	0.670	0.620	0.006*
75th percentile (HU)	90.00	0.800	0.625	0.696	0.660	0.001*
90th percentile (HU)	102.00	0.900	0.512	0.701	0.590	<0.001*
Kurtosis	3.04	0.650	0.662	0.656	0.660	0.027*
Area (cm^2)	438.59	0.600	0.837	0.731	0.790	0.002*
Max diameter (cm)	41.42	0.600	0.850	0.726	0.800	0.003*
SsD low	60.00	0.850	0.525	0.705	0.590	0.002*
Inertia GLCM 10	8.28	0.850	0.475	0.684	0.550	0.006*
Inertia GLCM 12	8.72	0.900	0.400	0.653	0.500	0.031*
Inertia GLCM 13	3.72	0.650	0.712	0.684	0.700	0.004*

AUC, area under the receiver operating characteristic (ROC) curve; GLCM, gray-level cooccurrence matrix; *p<0.05 with ROC curve analysis.

Table A3 Interobserver agreement for CT texture parameters based on the arterial phase

Parameters	ICC	Parameters	ICC
Mean	0.970	Histogram width	0.751
Standard deviation	0.760	Entropy GLCM 10	0.756
Max frequency	0.858	Entropy GLCM 11	0.752
Mode	0.921	Entropy GLCM 12	0.736
Minimum	0.820	Entropy GLCM 13	0.772
Maximum	0.928	Energy GLCM 10	0.749
5th percentile	0.901	Energy GLCM 11	0.763
10th percentile	0.917	Energy GLCM 12	0.733
25th percentile	0.949	Energy GLCM 13	0.790
50th percentile	0.971	Inertia GLCM 10	0.724
75th percentile	0.982	Inertia GLCM 11	0.837
90th percentile	0.985	Inertia GLCM 12	0.820
Skewness	0.621	Inertia GLCM 13	0.767
Kurtosis	0.461	Variance GLCM 10	0.774
Entropy	0.732	Variance GLCM 11	0.749
Area	0.896	Variance GLCM 12	0.743
Max diameter	0.926	Variance GLCM 13	0.742
SsD low	0.843		

ICC, intraclass correlation coefficient; GLCM, gray-level cooccurrence matrix.

Table A4 Interobserver agreement for CT texture parameters based on the venous phase

Parameters	ICC	Parameters	ICC
Mean	0.922	Histogram width	0.670
Standard deviation	0.627	Entropy GLCM 10	0.597
Max frequency	0.973	Entropy GLCM 11	0.607
Mode	0.836	Entropy GLCM 12	0.605
Minimum	0.714	Entropy GLCM 13	0.626
Maximum	0.896	Energy GLCM 10	0.565
5th percentile	0.846	Energy GLCM 11	0.589
10th percentile	0.879	Energy GLCM 12	0.583
25th percentile	0.905	Energy GLCM 13	0.635
50th percentile	0.920	Inertia GLCM 10	0.885
75th percentile	0.929	Inertia GLCM 11	0.890
90th percentile	0.942	Inertia GLCM 12	0.823
Skewness	0.393	Inertia GLCM 13	0.884
Kurtosis	0.579	Variance GLCM 10	0.601
Entropy	0.575	Variance GLCM 11	0.577
Area	0.935	Variance GLCM 12	0.572
Max diameter	0.870	Variance GLCM 13	0.579
SsD low	0.688		

ICC, intraclass correlation coefficient; GLCM, gray-level cooccurrence matrix.
